# Cellular Growth and Mitochondrial Ultrastructure of *Leishmania* (*Viannia*) *braziliensis* Promastigotes Are Affected by the Iron Chelator 2,2-Dipyridyl

**DOI:** 10.1371/journal.pntd.0002481

**Published:** 2013-10-17

**Authors:** Camila Mesquita-Rodrigues, Rubem F. S. Menna-Barreto, Leonardo Sabóia-Vahia, Silvia A. G. Da-Silva, Elen M. de Souza, Mariana C. Waghabi, Patrícia Cuervo, José B. De Jesus

**Affiliations:** 1 Laboratório de Pesquisa em Leishmaniose, Instituto Oswaldo Cruz, FIOCRUZ, Rio de Janeiro, Brasil; 2 Laboratório de Biologia Molecular e Doenças Endêmicas, Instituto Oswaldo Cruz, FIOCRUZ, Rio de Janeiro, Brasil; 3 Laboratório de Biologia Celular, Instituto Oswaldo Cruz, FIOCRUZ, Rio de Janeiro, Brasil; 4 Departamento de Microbiologia e Imunologia, Faculdade de Ciências Médicas, Universidade do Estado do Rio de Janeiro, Rio de Janeiro, Brasil; 5 Laboratório de Genômica Funcional e Bioinformática, Instituto Oswaldo Cruz, FIOCRUZ, Rio de Janeiro, Brasil; 6 Departamento de Engenharia de Biossistemas, Universidade Federal de São João de Rei, São João de Rei, Minas Gerais, Brasil; McGill university, Canada

## Abstract

**Background:**

Iron is an essential element for the survival of microorganisms *in vitro* and *in vivo*, acting as a cofactor of several enzymes and playing a critical role in host-parasite relationships. *Leishmania (Viannia) braziliensis* is a parasite that is widespread in the new world and considered the major etiological agent of American tegumentary leishmaniasis. Although iron depletion leads to promastigote and amastigote growth inhibition, little is known about the role of iron in the biology of *Leishmania*. Furthermore, there are no reports regarding the importance of iron for *L. (V.) braziliensis*.

**Methodology/Principal Findings:**

In this study, the effect of iron on the growth, ultrastructure and protein expression of *L. (V.) braziliensis* was analyzed by the use of the chelator 2,2-dipyridyl. Treatment with 2,2-dipyridyl affected parasites' growth in a dose- and time-dependent manner. Multiplication of the parasites was recovered after reinoculation in fresh culture medium. Ultrastructural analysis of treated promastigotes revealed marked mitochondrial swelling with loss of cristae and matrix and the presence of concentric membranar structures inside the organelle. Iron depletion also induced Golgi disruption and intense cytoplasmic vacuolization. Fluorescence-activated cell sorting analysis of tetramethylrhodamine ester-stained parasites showed that 2,2-dipyridyl collapsed the mitochondrial membrane potential. The incubation of parasites with propidium iodide demonstrated that disruption of mitochondrial membrane potential was not associated with plasma membrane permeabilization. TUNEL assays indicated no DNA fragmentation in chelator-treated promastigotes. In addition, two-dimensional electrophoresis showed that treatment with the iron chelator induced up- or down-regulation of proteins involved in metabolism of nucleic acids and coordination of post-translational modifications, without altering their mRNA levels.

**Conclusions:**

Iron chelation leads to a multifactorial response that results in cellular collapse, starting with the interruption of cell proliferation and culminating in marked mitochondrial impairment in some parasites and their subsequent cell death, whereas others may survive and resume proliferating.

## Author Summary

American tegumentary leishmaniasis (ATL) is a neglected disease that is widely distributed in the Americas. The protozoan parasite *Leishmania (Viannia) braziliensis* is one of the main causative agents of ATL, being responsible for the development of different clinical manifestations of the disease, which ranges from self-healing cutaneous lesions to disseminated and mucocutaneous forms. Because iron is essential for the survival and growth of *Leishmania*, as it is required for colonization of macrophages and development of lesions in mice, several chelating compounds have been tested for their effects on the growth of these parasites. In the present work, treatment of *L. (V.) braziliensis* with the iron chelator 2,2-dipyridyl inhibited the growth of promastigote forms in a dose- and time-dependent manner. However, multiplication of the parasites was recovered after reinoculation in fresh culture medium. The iron chelator also induced mitochondrial dysfunction and altered expression of proteins involved in metabolism of nucleic acids and coordination of post-translational modifications. The events described above ultimately caused the death of some parasites, most likely due to mitochondrial dysfunction, whereas others adapted and survived, suggesting a plasticity or resilience of the mitochondrion in this parasite.

## Introduction

*Leishmania (Viannia) braziliensis* is a protozoan parasite widely distributed in the New World. This species is considered the main etiological agent of American tegumentary leishmaniasis (ATL) [1] and has been associated with an extensive clinical polymorphism, ranging from simple cutaneous lesions to disseminated [2] and mucosal forms [3]. Like most living organisms, *Leishmania* require iron for their growth and survival. In these parasites, proteins involved in detoxification of reactive oxygen species, fatty acid desaturation and ergosterol synthesis have iron as a cofactor. Among those proteins, iron superoxide dismutase (SOD), ascorbate peroxidase (APX), cytochrome b5 (CytB5) and cytochrome p450 (CYP) are the most studied [4], [5]. In addition, iron is a component of ribonucleotide reductase and several heme-proteins and iron-sulfur clusters of the mitochondrial respiratory chain [5], [6]. Thus, iron also plays an essential role in energy metabolism and DNA synthesis [7].

Promastigote forms of *Leishmania* can acquire iron from transferrin [8], lactoferrin [9] and hemoglobin [10], . However, amastigotes express a ferrous iron transporter (LIT1) that is essential for the intracellular growth of parasites and development of cutaneous lesions in mice [12]. Recently, the gene that codes for *Leishmania* ferric reductase 1 (LFR1) was identified in *L (L.) amazonensis* [13]. LFR1 is a membrane protein with ferric reductase activity that is essential for conversion of extracellular Fe^+3^ into the soluble Fe^+2^, which is then transported into the amastigotes by LIT1. The ferric reductase activity of LFR1 can be detected on the cell surface of several *Leishmania* species and is required for the differentiation of *L. (L.) amazonensis* into metacyclic forms capable of initiating infections in the mammalian host [13].

Withdrawal of iron from the culture medium by either depletion of transferrin from fetal bovine serum (FBS) or removal of FBS from the medium inhibits the proliferation of *L. (L.) chagasi* promastigotes [9]. Depletion of iron by chelators affects growth and metabolism in several protozoan parasites. Incubation of *L. (L.) major* promastigotes with iron-chelating compounds significantly suppresses parasite growth in a dose-response manner [14]. The iron chelator desferrioxamine (DFO) inhibits the growth of late trophozoites and primary schizonts of *Plasmodium falciparum in vitro* [15]. In *Trypanosoma brucei*, DFO decreases growth rate, DNA synthesis and oxygen consumption [16], [17]. Depletion of iron by 2,2-dipyridyl, reduces the growth rate, adhesin synthesis and cytoadherence of *Trichomonas vaginalis* [18] and *Tritrichomonas foetus* [19]. Moreover, withdrawal of iron from the culture medium inhibits growth and induces drastic changes in the ultrastructure and proteomic pattern of *T. vaginalis* [20].

Despite the significance of iron for the growth, survival and establishment of infection by *Leishmania*, little is known about the role of this metal in the biology of the parasite. In this study we analyzed the effect of iron depletion on the proliferation, ultrastructure and protein expression pattern of *L. (V.) braziliensis*. The results show that iron depletion leads to a multifactorial response that results in cellular collapse, starting with the interruption of cell proliferation and culminating in marked mitochondrial impairment in some parasites and their subsequent cell death, whereas others may survive and resume proliferating.

## Methods

### Ethics statement

The *L. (V.) braziliensis* isolate IOC-L 2483 (MHOM/BR/2000/LTCP 13396) used in this study was obtained from the *Leishmania* collection of the Oswaldo Cruz Institute (Coleção de *Leishmania* do Instituto Oswaldo Cruz, CLIOC) (http://clioc.fiocruz.br/). CLIOC is registered in the World Federation for Culture Collections (WFCC-WDCM 731) and is recognized as a Depository Authority by the Brazilian Ministry of the Environment (D.O.U. 05.04.2005).

### Chemicals

All reagents were purchased from Sigma (St. Louis, MO, USA) or Merck (São Paulo, SP, Brazil). Milli-Q-purified water (Millipore Corp., Bedford, MA, USA) was used to make all solutions. The iron chelator 2,2-dipyridyl is an organic, synthetic, membrane-permeable compound that associates with extracellular and intracellular iron, preferentially binding Fe^+2^ ions that constitute the cytosolic labile iron pool [21]–[23].

### Parasite culture and proliferation

Promastigotes from *L. (V.) braziliensis* strain IOC-L 2483 were grown at 25°C in Schneider's medium supplemented with 20% (v/v) FBS (heat-inactivated at 56°C for 50 min). To evaluate the influence of iron chelation on parasite proliferation, 1×10^6^ parasites were inoculated in fresh Schneider's medium in the absence (control) or presence of 25, 50, 100, 140 or 180 µM 2,2-dipyridyl and incubated at 25°C. Cellular density was determined every 24 h for eight days by counting with a hemocytometer. Three independent assays were carried out in triplicate.

### Cytotoxicity of iron chelator on *L. (V.) braziliensis*

To evaluate the cytotoxicity of 2,2-dipyridyl on promastigote forms, 2×10^6^ parasites were resuspended in 1 ml of Schneider's medium plus 20% FBS. This suspension (100 µl) was added to 80 µl of 2,2-dipyridyl solution to obtain final concentrations of 25–180 µM. Parasites (2×10^5^/well) were incubated in 96-well plates at 25°C for 16 or 40 h. After this period, 20 µl of resazurin (Invitrogen, OR, USA) was added to each well. Resazurin is a non-fluorescent, cell-permeable compound. After entering viable cells, which maintain a reducing environment within the cytosol, resazurin is continuously converted to resorufin, a highly fluorescent compound. After 8 h of incubation, fluorescence emitted due to conversion of resazurin into resorufin was detected at 570 nm (excitation peak) and 585 nm (emission peak). After the cytotoxicity assays, the results were plotted in graphs showing the fluorescence intensity versus the concentration of 2,2-dipyridyl. The inhibitory concentrations responsible for 50% reduction in cell viability (IC_50_) were obtained after 24 and 48 h of treatment. Student's T test was used to compare promastigotes grown in control and iron-depleted medium. Differences with p≤0.01 were considered statistically significant.

### Analysis of the reversibility of the effect of 2,2-dipyridyl on parasite proliferation

For this assay, 1×10^6^ promastigotes at the logarithmic phase of growth were cultivated for 24 or 48 h in the presence or absence of 100 µM iron chelator. Subsequently, the parasites were washed 2× with PBS and inoculated in fresh Schneider's medium plus 20% FBS, and cell density was determined daily for eight days by counting with a hemocytometer. As an additional control, another sample of parasites was continuously cultured in the presence of 100 µM for four days. Three independent assays were carried out in triplicate.

### Transmission electron microscopy (TEM) analysis

Promastigotes (5×10^6^ cells/ml) were treated with 100 µM 2,2-dipyridyl for 24 h in Schneider medium at 25°C. Afterwards, the parasites were fixed with 2.5% glutaraldehyde in 0.1 M Na-cacodylate buffer (pH 7.2) at room temperature for 40 min at 25°C and post-fixed with a solution of 1% OsO_4_, 0.8% potassium ferricyanide and 2.5 mM CaCl_2_ in the same buffer for 20 min at 25°C. The cells were dehydrated in an ascending acetone series and embedded in PolyBed 812 resin. Ultrathin sections were stained with uranyl acetate and lead citrate and examined in a JEOL JEM1011 transmission electron microscope (Tokyo, Japan).

### Mitochondrial membrane potential (ΔΨm) and plasma membrane integrity analysis

Promastigote forms of *L. (V.) braziliensis* were treated with 25, 50 or 100 µM 2,2-dipyridyl for 24 h or 48 h and subsequently evaluated for (a) the mitochondrial membrane potential (ΔΨm) or (b) the plasma membrane integrity. To assess the ΔΨm, the parasites were incubated with 50 nM tetramethylrhodamine (TMRE) (Molecular Probes, Carlsbad, USA) for 30 min. Alterations in TMRE fluorescence were quantified using an index of variation (IV) obtained by the equation (MT - MC)/MC, where MT is the median fluorescence of treated parasites and MC, that of control parasites. Negative IV values correspond to depolarization of the mitochondrial membrane. As a positive control, 10 µM carbonyl cyanide 4-(trifluoromethoxy) phenylhydrazone (FCCP) (Sigma-Aldrich Chemical Co.), which dissipates the ΔΨm, was added. To evaluate the plasma membrane integrity, labeling with 30 µg/mL propidium iodide (PI) for 30 min was performed, using 0.1% saponin as the positive control. The samples were analyzed in a FACSCalibur flow cytometer (Becton Dickinson, CA, USA) equipped with CellQuest software (Joseph Trotter, Scripps Research Institute, La Jolla, USA). A total of 10.000 events were acquired in the region previously established as that of the parasites. The Mann-Whitney test was used to compare the control and treated groups. Differences with p≤0.05 were considered statistically significant.

### *In situ* labeling of DNA fragments by terminal deoxyribonucleotide transferase-mediated dUTP nick end labeling (TUNEL)

Promastigote forms of *L. (V.) braziliensis* (10^6^) were treated with 100 µM 2,2-dipyridyl for 24 h or 48 h, and *in situ* detection of DNA fragments was performed by TUNEL (Sigma-Aldrich Chemical Co). Promastigotes were centrifuged (3,000× *g*, 10 min, 4°C), resuspended in PBS, spotted onto slides previously treated with poly-L-lysine and air-dried. Then, they were fixed with 4% paraformaldehyde for 10 minutes and washed once with PBS. Subsequently, parasites were blocked with 0.03% H_2_O_2_ in methanol, washed in PBS and permeabilized with 0,1% Triton X-100 in 0,1% sodium citrate for 2 min at 4°C. Positive controls were treated with 12 µl of 1× DNase for 15 min and blocked with 25 mM EDTA for 10 min. Finally, promastigotes were incubated with TUNEL reaction mixture for 60 min at 37°C. Then, they were washed and incubated with 300 µM DAPI (Invitrogen) for 2 min. After labeling, the material was washed with PBS, and coverslips were mounted on slides containing n-propyl-gallate. Images were acquired by phase contrast and fluorescence microscopy.

### Sample solubilization and protein precipitation

Parasites (1×10^9^) treated or not with 100 µM 2,2-dipyridyl for 24 h were harvested by centrifugation at 3.000× *g* for 10 min at 4°C, washed three times in PBS pH 7.2 and lysed (by 15 cycles of freezing and thawing in liquid nitrogen and ultrasonication) in hypotonic PBS buffer (13.6 mM NaCl, 0.27 mM KCl, 0.4 mM Na_2_HPO_4_, 0.15 mM KH_2_PO_4_) containing a cocktail of protease inhibitors. The lysate was centrifuged at 16.000× *g* for 30 min at 4°C to remove insoluble material, and the proteins in the resulting supernatant were precipitated with 10% (v/v) TCA and washed with cold acetone at 16.000× *g* for 10 min. Finally, the pellet was resuspended in IEF buffer (9 M urea, 4% CHAPS, 65 mM dithiothreitol (DTT) and 1% ampholytes pH 3–10) for 1 h at room temperature. Protein concentration was determined using the 2D Quant Kit (GE Healthcare). Proteins were aliquoted into single-use samples of 500 µg and stored at −80°C until analysis.

### 2DE electrophoresis, protein visualization and image analysis

For the first dimension, 500 µg protein was diluted to a final volume of 350 µl in rehydration solution (9 M urea, 4% CHAPS, 65 mM DTT, 1.5% ampholytes pH 3–10, 0,001% bromophenol blue). This solution was applied to IEF strips (18 cm pH 3–10 nonlinear; GE Healthcare) and submitted to isoelectric focalization at Ettan IPGphor 3 (GE Healthcare) at 20°C and a maximum current of 50 µA/strip. Focusing parameters and the second dimension were set as previously described [24]. Detection of spots and comparison of protein expression were performed using PDQuest software (Bio-Rad). The intensity of each spot, measured in parts per million (ppm), provided the basis for comparison of protein expression in parasites cultured in control medium or treated with the iron chelator. To normalize the intensity values, the pixel intensity of each spot, measured in ppm, was divided by the total intensity of all pixels present in the image.

### Protein digestion, peptide extraction and analysis by mass spectrometry

Protein spots were manually excised and treated for digestion. Gel pieces were washed three times in 400 µL of 50% acetonitrile, 25 mM NH_4_HCO_3_ pH 8,0dehydrated in acetonitrile 100% and dried in a vacuum centrifuge. Gel pieces were rehydrated in 15 µl of 50 mM NH_4_HCO_3_ with 200 ng of trypsin (Promega). After 15 min, 20 µl of 50 mM a NH_4_HCO_3_ was added to keep gel pieces wet during tryptic digestion (37°C, overnight). To extract peptides, 20 µl of 0.5% (v/v) trifluoroacetic acid (TFA) in 50% (v/v) acetonitrile were added, and samples were sonicated for 30 min. The separated liquid was concentrated under a vacuum to an approximate volume of 10 µl. Tryptic peptides were purified using ZipTip C18 pipette tips following the manufacturer's instructions (Millipore), eluted with 3.0 µl of 0.1% (v/v) TFA in 50% (v/v) acetonitrile and co-crystallized with matrix (7 mg/mL alpha-cyano-4-hydroxycinnamic acid) on a stainless-steel plate using 0.5 µl of matrix and 0.5 µl of sample. Mass spectra were acquired on a 5800 Proteomics Analyzer mass spectrometer (MALDI-TOF/TOF™, Applied Biosystems) operating in delayed reflector mode with an accelerated voltage of 20 kV. MS/MS spectra corresponding to the five most intense signals were obtained automatically using the CID acquisition mode. Proteins were identified by searching in the non-redundant database of the National Center for Biotechnology Information (NCBInr) using the program Mascot MS/MS ion search (Matrix Science, Oxford, UK, www.matrixscience.com/search_form_select.html). The search parameters in the Mascot server were lack of taxonomic restrictions; permission of tryptic peptides with only one erroneous cleavage; carbamidomethylation of cysteine residues as a fixed modification and oxidation of methionine as a variable modification; 100 ppm mass tolerance for the MS mode; and 0.2 Da tolerance for its corresponding fragments in MS/MS. Proteins identified as hypothetical and therefore of unknown function were analyzed using InterProScan Sequence Search Tool (http://www.ebi.ac.uk/Tools/pfa/iprscan/) from the InterPro data library (http://www.ebi.ac.uk/Interpro/) to assign predicted functional domains [25]. This data library combines independent databases to generate an integrated source of information about protein families, domains, sites and regions, thus enhancing annotation. The data were analyzed following the standards proposed under the Minimum Information About a Proteomic Experiment MIAPE consensus [26].

### Gene expression analysis

Total RNA was extracted from the cells by using Trizol Reagent. cDNA was synthesized from 2 µg total RNA using a commercial reverse transcription system (Promega). qPCR was performed in duplicate using the SYBR Green PCR Master Mix in Step-One equipment according to the manufacturer's protocol. Primers for amplifying the target and reference genes *EIF5A* (eukaryotic initiation factor 5A), *CAL* (calmodulin A), *UCEE2* (ubiquitin-conjugated enzyme E2), *UCELP* (ubiquitin-conjugating enzyme-like protein), *RP18* (ribonucleoprotein p18 mitochondrial precursor), *60S* (60S acid ribosomal protein P2) and *ACT* (actin) were designed based on the genome sequences available for *L. (V.) braziliensis* (Table S1). The data are shown as normalized ratios between the target gene expression and the reference gene [27]. Experiments were performed following the MIQE guidelines [28].

## Results

### Effect of iron chelator on proliferation of *L. (V). braziliensis* promastigotes *in vitro*

**Figure 1:**
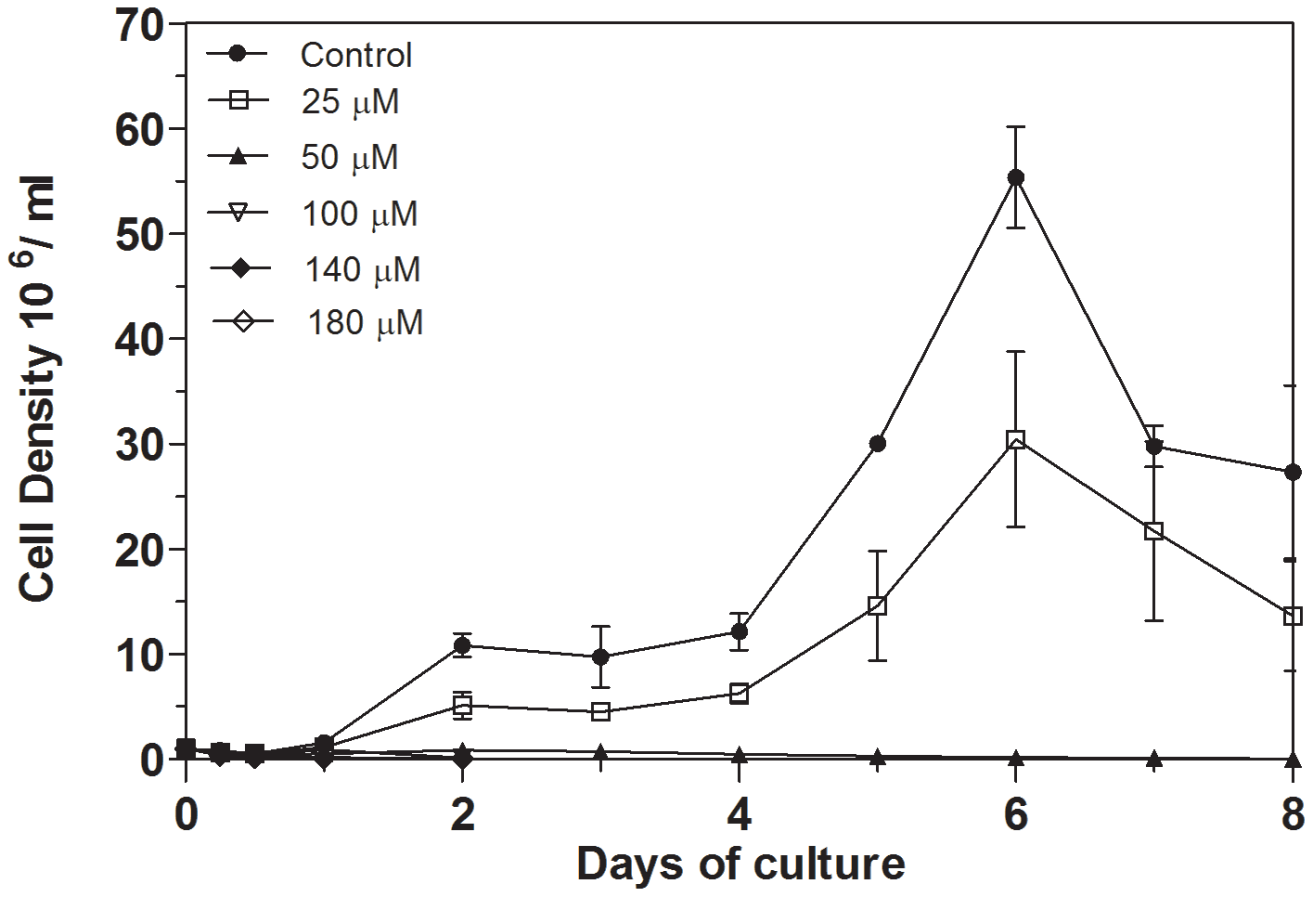
Effect of iron depletion on the growth curve of *L. (V.) braziliensis* promastigotes. Parasites were cultivated at 25°C in Schneider's medium supplemented with 20% of fetal bovine serum (control) or FBS-medium treated with 25, 50, 100, 140 or 180 µM 2,2-dipyridyl. The growth curve was monitored daily for eight days. Counts were performed in a hemocytometer. Bars represent means and standard errors obtained from three independent experiments.

To determine the influence of iron chelation on the growth of *L. (V.) braziliensis*, 1×10^6^ promastigotes were incubated in 10 ml of Schneider's medium containing different concentrations of 2,2-dipyridyl (Figure 1). Cell density was measured daily for eight days of culture by counting in a hemocytometer chamber. In the control medium, promastigotes reached the late logarithmic phase of growth after six days. In the presence of 25 µM of the chelator, promastigote proliferation was reduced at all points of the curve. However, the growth curve profile was similar to that displayed by parasites grown in the control medium, reaching the logarithmic phase of growth at the sixth day. Treatment with 50–180 µM iron chelator resulted in a drastic inhibition of cell proliferation (Figure 1).

### Cytotoxicity of 2,2-dipyridyl to *L. (V.) braziliensis*

**Figure 2:**
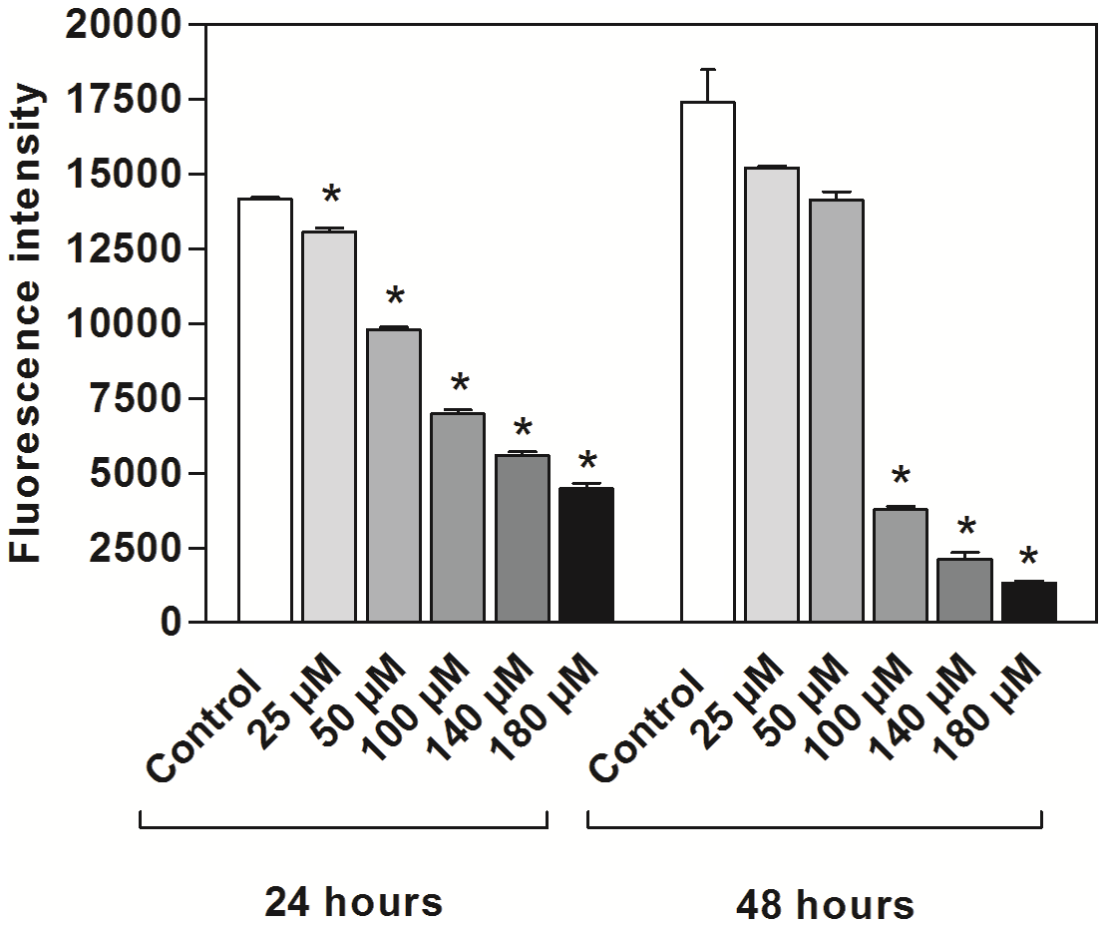
Cytotoxicity of 2,2-dipyridyl to *L. (V.) braziliensis* after 24 and 48 h. Parasites were incubated at 25°C in Schneider's medium in the absence (control) or presence of 25, 50, 100, 140 or 180 µM 2,2-dipyridyl. The graphs illustrate the relationship between time of incubation and fluorescence emitted after the reduction of resazurin. Bars represent means and standard errors obtained from three independent experiments. Asterisks represent a significant reduction in fluorescence after treatment of parasites in relation to the experimental control (p<0.01, Student's T test).

The cytotoxicity of the iron chelator to the promastigote forms was assessed after 24 and 48 h using the Alamar Blue redox indicator. There was a dose-dependent relationship between the fluorescence emitted by reduction of resazurin and the concentration of the iron chelator. Concentrations of 100, 140 and 180 µM significantly affected the fluorescence emitted after 24 and 48 h of incubation compared with controls (p<0.01) (Figure 2). We also observed a correlation between fluorescence and time of exposure to the iron chelator. In promastigotes treated with 100, 140 or 180 µM of the iron chelator, the values of fluorescence emitted after 48 h of incubation were lower than those emitted after 24 h. On the other hand, fluorescence emitted by reduction of resazurin was higher after 48 h of culture in the control medium. The same was observed for parasites treated with 25 or 50 µM of 2,2-dipyridyl (Figure 2). Based on the relationship between the concentration of the iron chelator and the fluorescence emitted, the IC_50_ of 2,2-dipyridyl was 100 µM and 83.2 µM for 24 and 48 h, respectively.

### Recuperation of parasite proliferation after treatment with 2,2-dipyridyl

**Figure 3:**
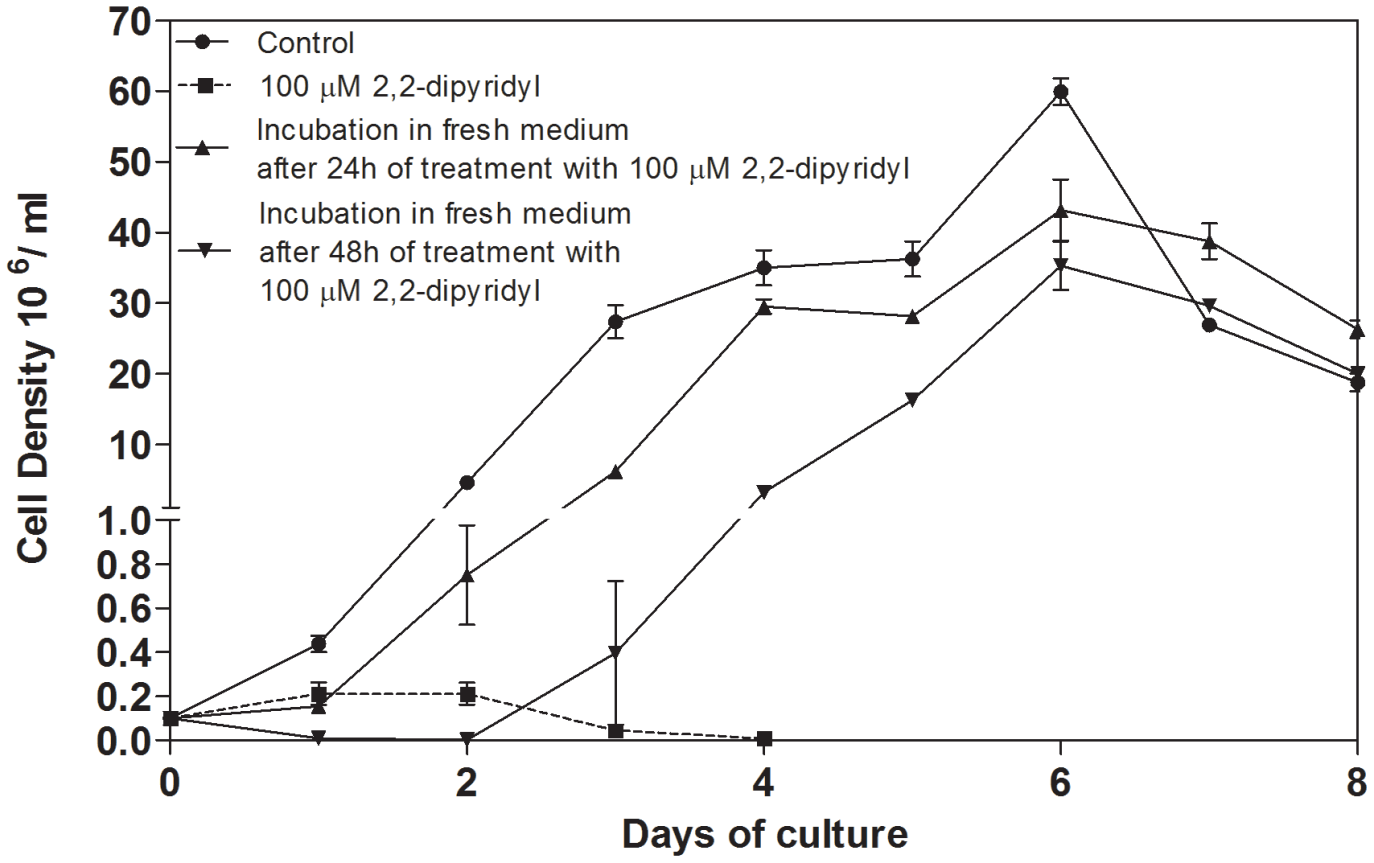
Reversibility of the effect of 2,2-dipyridyl on *L. (V.) braziliensis* proliferation. Parasites were incubated with 100 µM iron chelator and resuspended in Schneider's fresh medium after 24 h or 48 h of treatment. Cell density was determined daily for eight days by counting with a hemocytometer. Proliferation of parasites cultivated in the control medium as well as the growth of parasites continually cultured in the presence of 100 µM 2,2-dipyridyl were also monitored. Bars represent means and standard errors obtained from three independent experiments.

To determine whether the multiplication of *L. (V.) braziliensis* could be recovered after treatment with 2,2-dipyridyl, parasites treated with 100 µM 2,2-dipyridyl were re-inoculated in fresh control medium and followed daily for eight days. Parasites treated for 24 h or 48 h with the chelator recovered their ability to proliferate after being placed in fresh Schneider's medium, reaching the stationary phase of growth on the same day as control parasites (Figure 3). The chelator-treated parasites exhibited slightly impaired proliferation during the early stage of growth (days 1–3.5), which reflected their re-adaptation to the fresh medium (Figure 3).

### Effect of 2,2-dipyridyl on the ultrastructure of *L. (V.) braziliensis*

**Figure 4:**
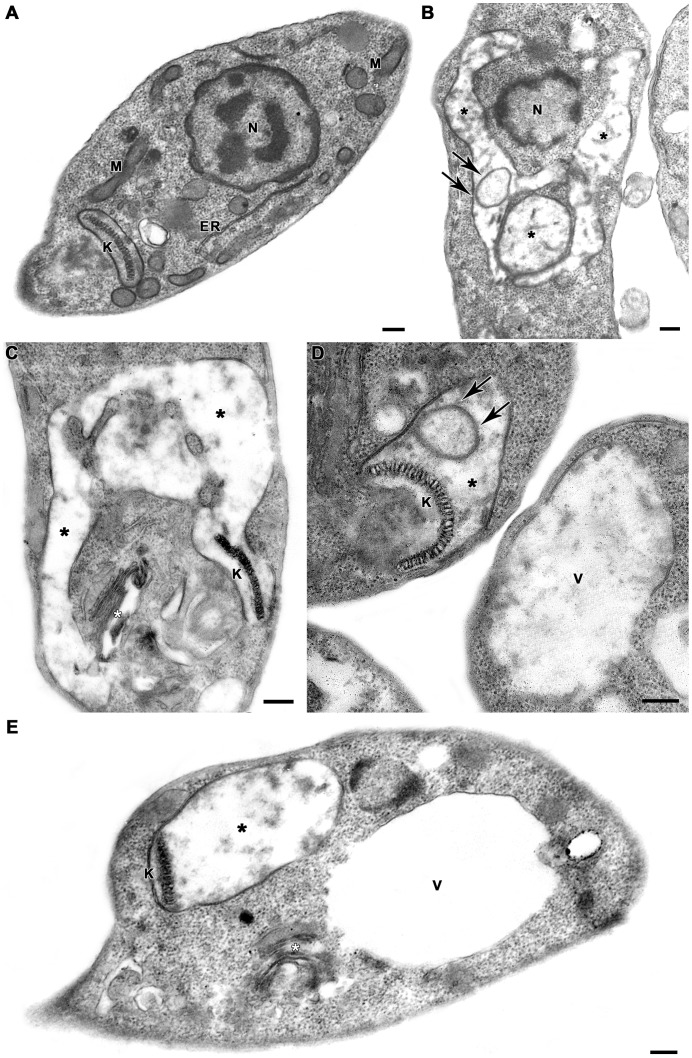
Ultrastructural effects of 2,2-dipyridyl on *L. (V.) braziliensis* promastigotes. (A) Untreated parasite presenting typical elongated morphology with normal kinetoplast (K), mitochondrion (M), endoplasmic reticulum (ER) and nucleus (N). (B–E) Promastigotes treated with 100 µM 2,2-dipyridyl revealed marked mitochondrial swelling with loss of cristae and matrix (black asterisks). There were concentric membranar structures inside the organelle (arrows), without any impairment of the classic kDNA arrangement. The treatment with 100 µM 2,2-dipyridyl also induced Golgi disruption (white asterisks) and extensive cytoplasmic vacuolization (V). Bars = 200 nm.

Ultrastructural analysis revealed severe damage to the parasite mitochondrion (Figure 4). Promastigotes treated with 2,2-dipyridyl presented mitochondrial swelling with concentric membranar structures inside the organelle (Figure 4B, D). Severe loss of cristae and matrix were also seen. Moreover, the matrix displayed a washed-out appearance, indicating a decrease in the electron density. (Figure 4B–E). Treatment with the iron chelator also induced Golgi disruption (Figure 4C, E) and extensive cytoplasmic vacuolization (Figure 4D–E). No damage to kinetoplast DNA (Figure 4C–E) or nuclear DNA was observed (Figure 4B).

### Mitochondrial membrane potential (ΔΨm) and plasma membrane integrity analysis

**Figure 5:**
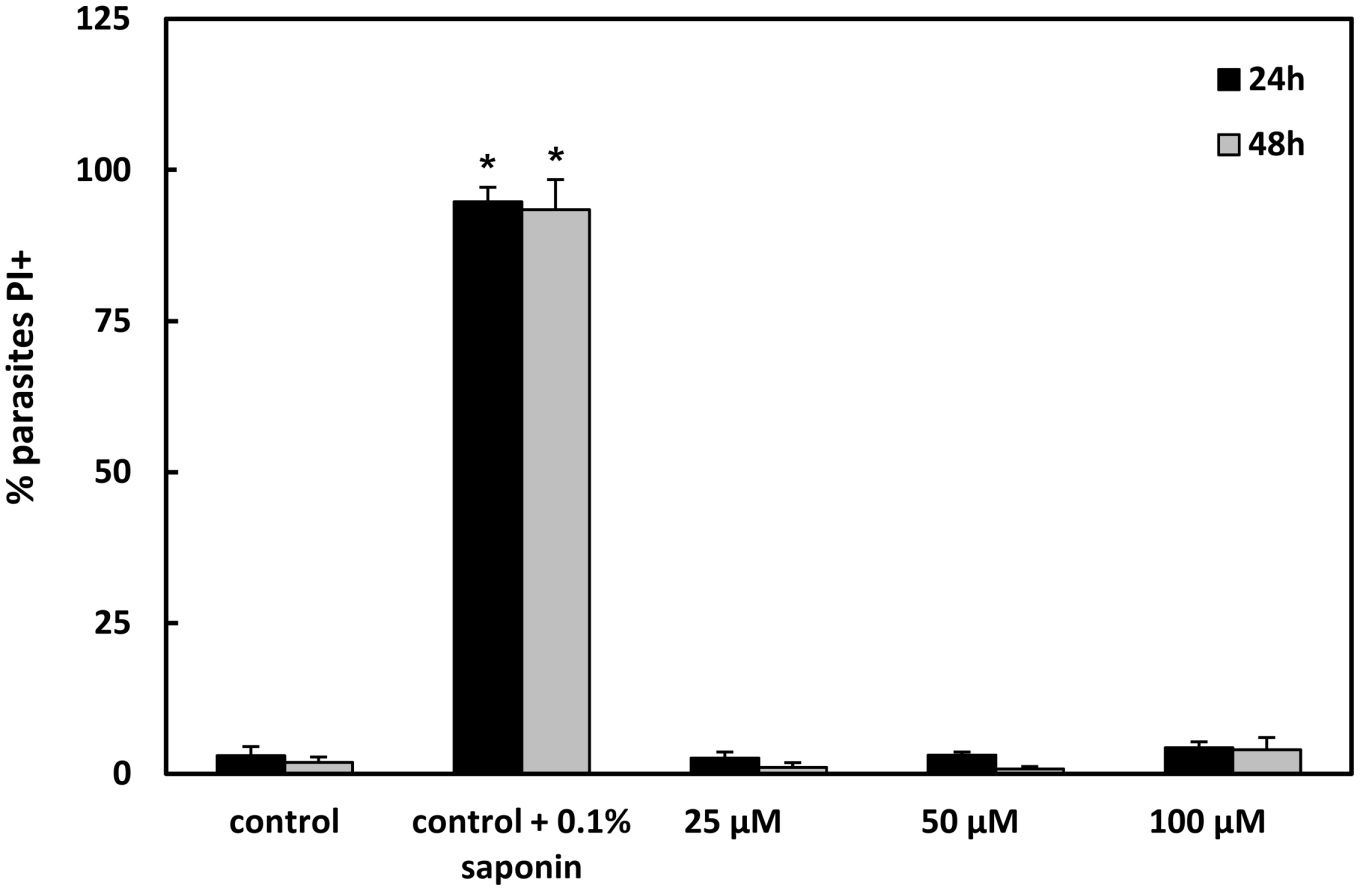
Flow cytometry analysis of *L. (V.) braziliensis* promastigotes' plasma membrane integrity after 24 and 48 h. Parasites were incubated at 25°C in Schneider's medium in the absence (control) or presence of 25, 50 or 100 µM 2,2-dipyridyl. In addition, control parasites were treated with 0.1% saponin. The samples were labeled with labeling with 30 µg/mL propidium iodide (PI) and analyzed in a FACSCalibur flow cytometer. Mean ± standard deviation of 3 independent experiments. Asterisks indicate significant differences in relation to the control (untreated) group at each time (* p≤0.05).

Flow-cytometric analysis showed that 2,2-dipyridyl led to a dose-dependent decrease in the ΔΨm in comparison to control parasites (Table 1). Treatment with 25, 50 and 100 µM iron chelator induced a TMRE fluorescence reduction of 9%, 28% and 40% at 24 h and 7%, 2% and 25% at 48 h, respectively. Incubation of the cells with 10 µM FCCP dissipated the ΔΨm and consequently decreased the extent of TMRE labeling, strongly indicating the mitochondrial specificity of the TMRE labeling. The percentage of PI-positive cells was similar in control and treated parasites at 24 and 48 h, indicating that the plasma membrane of treated promastigotes did not suffer permeabilization (Figure 5).

**Table 1:** Flow cytometry analysis of ΔΨm and plasma membrane integrity in *L. (V.) braziliensis* promastigotes.

	% TMRE+		Index of Variation (IV)^1^	
	24 h	48 h	24 h	48 h
Control	96.0±1.1^2^	98.0±0.7	0.00	0.00
control + 10 µM FCCP	12.0±8.2*	32.1±5.3*	−0.74	−0.52**
25 µM 2,2-dipyridyl	95.6±2.4	94.6±3.8	−0.09**	−0.07
50 µM 2,2-dipyridyl	93.3±3.8	96.4±3.2	−0.28**	−0.02
100 µM 2,2-dipyridyl	89.3±6.3	73.9±12.0*	−0.40**	−0.25**

TMRE+ = promastigotes labeled by tetramethylrhodamine. FCCP = carbonyl cyanide 4-(trifluoromethoxy) phenylhydrazone.

1 IV = (MT – MC)/MC, where MT corresponds to the median TMRE fluorescence of treated parasites and MC corresponds to the median TMRE fluorescence of control parasites.

2 Mean ± standard deviation of 4 independent experiments. Asterisks indicate significant differences in relation to the control group at each time (* p≤0.01; ** p≤0.03).

### Analysis of DNA fragmentation in *L. (V.) braziliensis* after treatment with iron chelator

**Figure 6:**
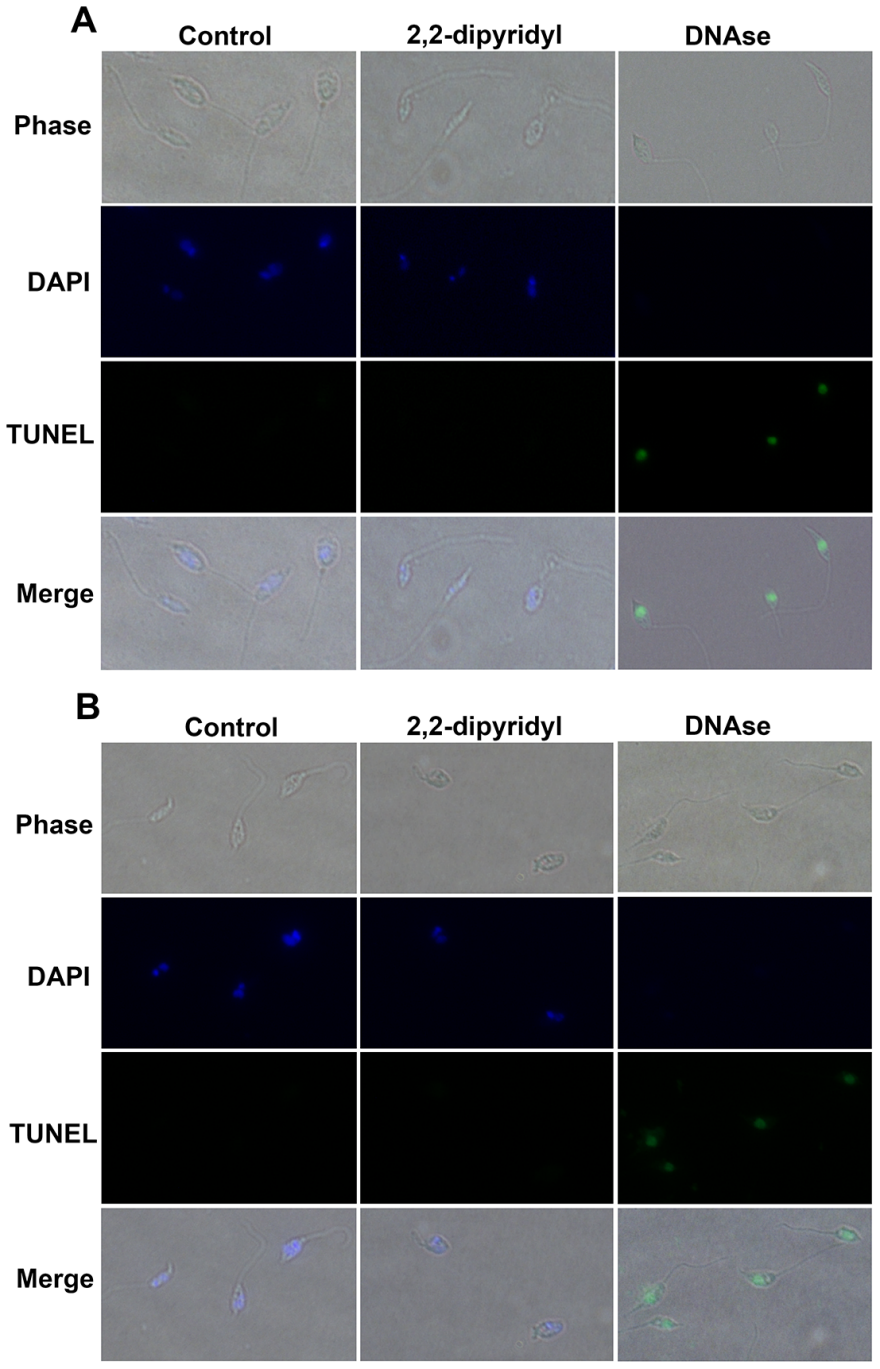
TUNEL assay. Promastigotes cultivated in control medium or treated with 100 µM 2,2-dipyridyl for 24 h (A) or 48 h (B) were examined by phase contrast microscopy and fluorescence microscopy at 100×. DAPI (blue channel) revealed intact nuclei and kinetoplasts in both control and treated promastigotes. TUNEL (green channel) detected DNA fragmentation exclusively in promastigotes treated with DNase. Merged images show the localization of the nuclei and kinetoplasts inside the cells.

DNA of promastigotes grown for 24 h or 48 h in the presence or absence of 2,2-dipyridyl was labeled exclusively by DAPI (Figure 6), indicating that treatment with the iron chelator did not induce DNA fragmentation. Because DNase induces DNA strand degradation, promastigotes treated with DNase were used as positive controls for the TUNEL assay. Promastigotes treated with DNase were labeled by TUNEL but not by DAPI (Figure 6).

### Effect of 2,2-dipyridyl on the protein expression of *L. (V.) braziliensis* and on the mRNA levels of differentially expressed proteins

**Figure 7:**
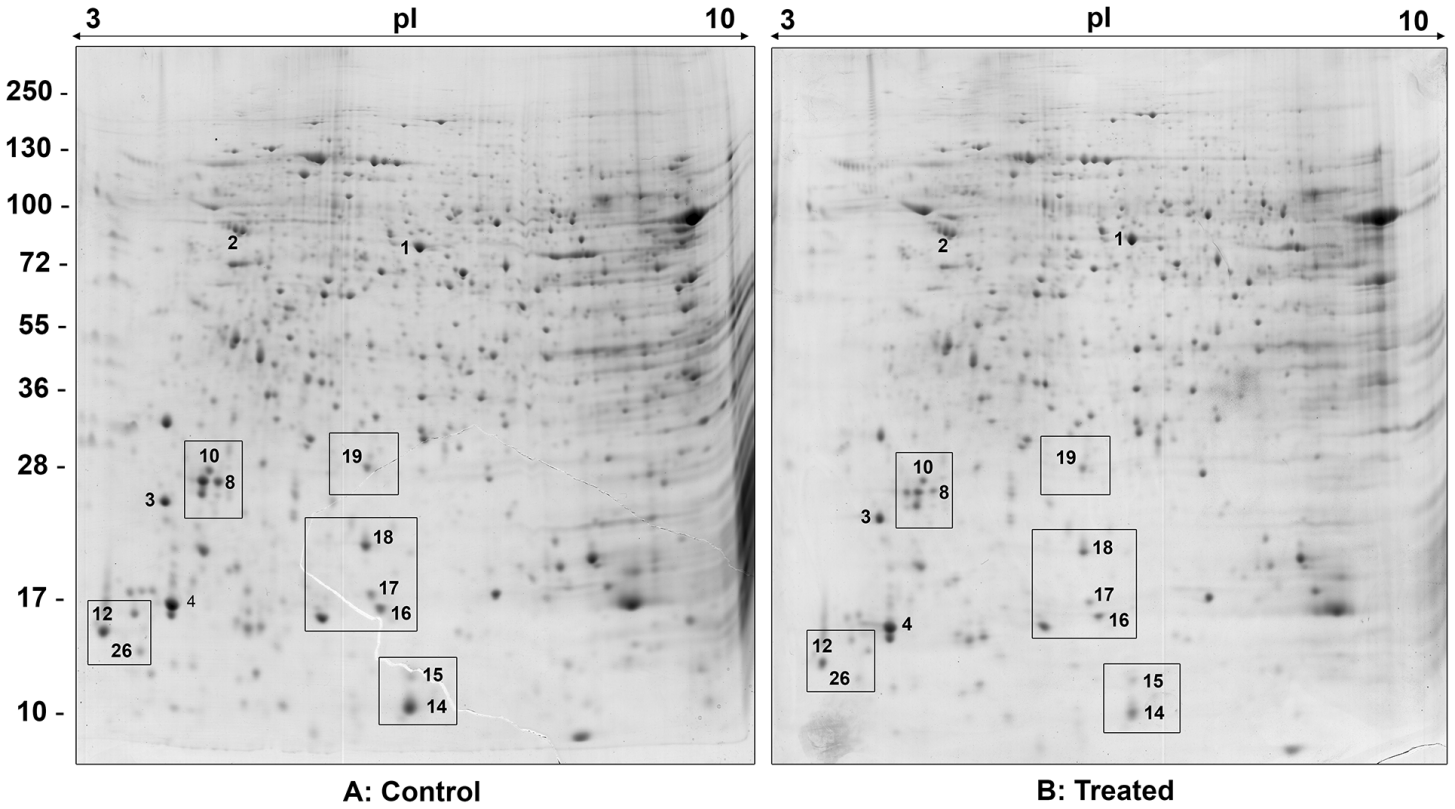
2DE map of soluble proteins from promastigotes cultivated in (A) control medium or (B) medium with 2,2-dipyridyl. Proteins were separated over a pH range of 3–10 in 12% SDS-polyacrylamide gel. Protein spots were visualized by colloidal Coomassie Blue G-250 staining. Differentially expressed proteins are numbered, and details of their identification are shown in Table 2 and Table S2.

**Figure 8:**
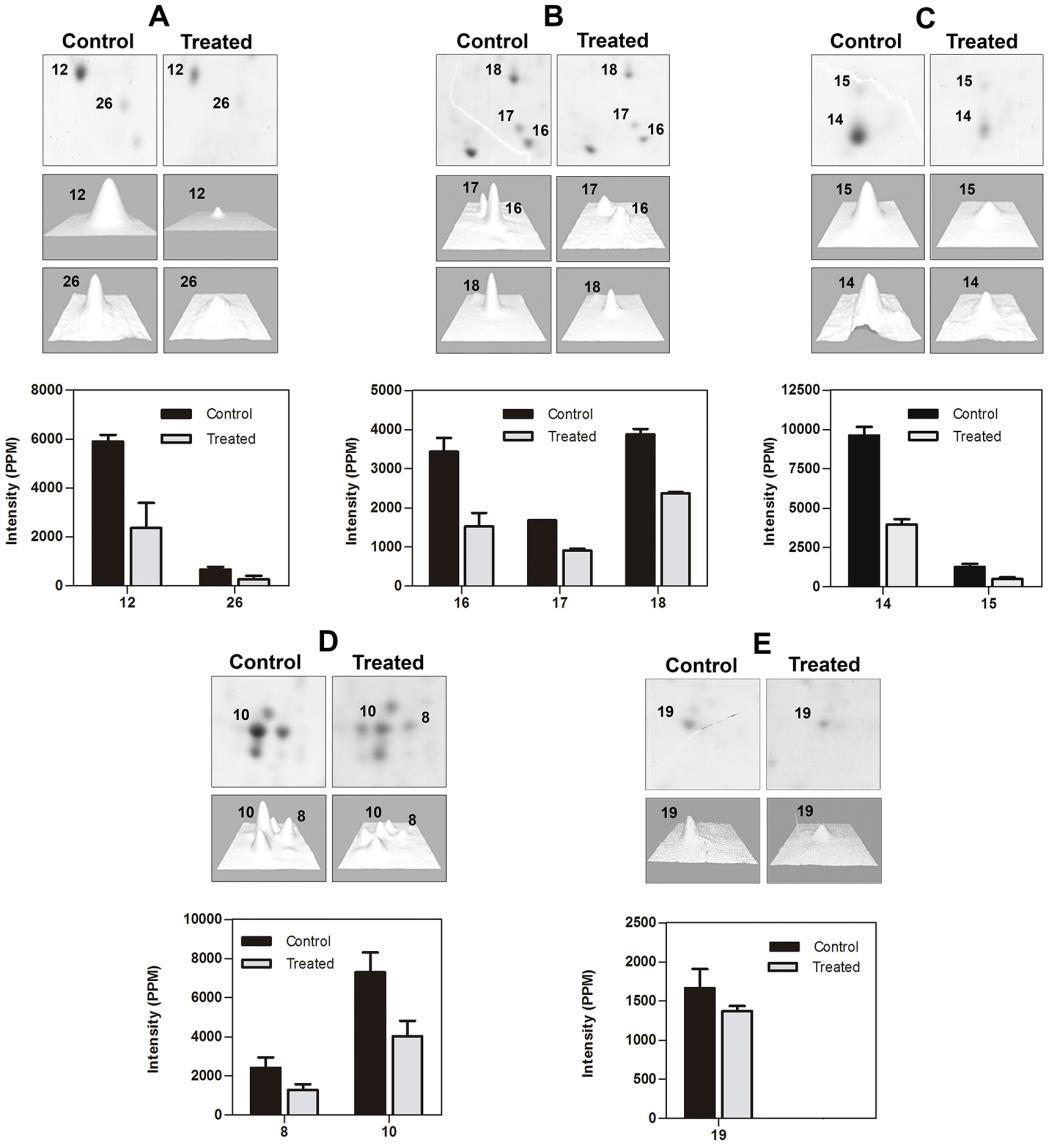
Close-up of the gel regions showing differentially expressed proteins in control and treated parasites. (A–E) Close-ups of different regions of the gels showing up-regulated and down-regulated proteins. The degree of differential expression is shown in the histograms, presented as a grouped bar chart with error bars. Each bar represents the intensity means ± S.D. of gels from three independent experiments.

To investigate the effect of 2,2-dipyridyl on protein expression, whole extracts from parasites grown in control medium or treated with the iron chelator were submitted to 2DE (Figure 7). Image analysis was performed by comparing representative gels obtained from three different parasite suspensions for each condition assayed. The spots that showed a twofold (2×) increase or decrease in pixel intensity were considered differentially expressed (Figure 7, Table 2). In addition, to further confirm that these pixel intensity differences represented distinct expression levels, we also characterized spots that were invariant between control and chelator-treated parasites (Figure S1, Table S2). Nine protein spots were down-regulated in parasites grown in the presence of the iron chelator (Figure 7, Table 2). Comparative close-ups of different regions of the gels show the proteins that were differentially expressed between parasites cultivated in the presence and in the absence of the iron chelator (Figure 8 A–E). The proteins are numbered according to Figure 7. The intensity of each differentially expressed spot is represented in the graphs and histograms arranged together with the close-ups (Figure 8 A–E). Although spot 19, corresponding to cytochrome *c* oxidase, showed a less than twofold decrease in the chelator-treated parasites, it was included in the differential analysis because a subtle decrease in the expression of this protein could have substantially contributed to the mitochondrial dysfunction observed here (Figure 8E). The biological and/or molecular function of the differentially expressed proteins was inferred from the terms of gene ontology (GO) available in TriTrypDB (http://tritrypdb.org/tritrypdb/) or using InterProScan Sequence Search (http://www.ebi.ac.uk/Tools/pfa/iprscan/) (Table 2). The differentially expressed proteins were associated with nucleic acid metabolism, calcium homeostasis, signaling and post-translational modifications of proteins. To determine whether the differential expression of proteins induced by 2,2-dipyridyl resulted from modulation at the mRNA level, primers were designed for the gene encoding each protein, and qPCR was performed. The results show that there was no difference in the mRNA expression level of any protein (Figure S2).

**Table 2:** Proteins differentially expressed by *L. (V.) braziliensis* promastigotes cultivated in control or chelator-treated medium.

Code	Protein Name	NCBI Accession N°	Theor. MW (Exp. MW)	Theor. p*I* (Exp p*I*)	Matching pep./Pep. identified by MS/MS	Peptide Sequences	Error ± ppm	Protein Score	Expression in control medium	Expression in chelator-treated medium	BP, CC and MF
8	Chain A, *Leishmania braziliensis* Eukaryotic Initiation Factor 5a	1X6O_A	18.93	5.42	3/2	TYPLAAGALK	−50	215	+	−	BP: Regulation of translational elongation; CC: MF: RNA binding
			(27)	(5.21)		VSIVATDIFTGNR	−45				
						LEDQAPSTHNVEVPFVK	−49				
10	Chain A, *Leishmania braziliensis* Eukaryotic Initiation Factor 5a	1X6O_A	18.93	5.42	3/3	TYPLAAGALK	−64	295	+	−	BP: Regulation of translational elongation; CC: MF: RNA binding
			(27)	(5.1)		VSIVATDIFTGNR	−58				
						LEDQAPSTHNVEVPFVK	−71				
12	calmodulin A *Trypanosoma brucei*	AAA30174	16.82	4.10	6/6	A▪DQLSNEQISEFK	−94	546	+	−	CC: Flagellum; MF: Calcium ion binding
			(13.5)	(4.2)		EAFSLFDKDGDGTITTK	−95				
						VFDKDGNGFNSAAELR	−57				
						DGNGFNSAAELR	−51				
						LTDEEVDEM ^▴^ IR	−89				
						EADVDGDGQINYEEFVK	−95				
14	hypothetical protein *Leishmania braziliensis*	XP_001563170	13.30	5.71	5/5	KFGYVDYTK	−84	365	+	−	ND
			(10)	(5.98)		FGYVDYTK	−81				
						AQFDDVR	−54				
						STTTDKIEVTVVK	−91				
						SPDFDAIYEQQQK	−91				
15	hypothetical protein *Leishmania infantum*	XP_001467133	13.69	5.9	1/1	FIFLSNPDHWR	−48	60	+	−	ND
			(12)	(5,97)							
16	ubiquitin conjugated enzyme E2 *Trypanosoma cruzi*	XP_812602	16.87	6.08	1/1	VLLSVCSLLTDPNPDDPLVPDIAR	−66	77	+	−	BP: regulation of protein metabolic process
			(14.5)	(5.84)							
17	ubiquitin-conjugating-enzyme like protein *Leishmania braziliensis*	XP_001563284	16.26	5.93	2/2	DTEDIYFHYWNGTIVGPPSSTFEYR	−52	59	+	−	BP: regulation of protein metabolic process MF: post-translational protein modification
			(15.2)	(5.81)		VNLPCVDPDGTVNR	−81				
18	ribonucleoprotein p18 mitochondrial	XP_001563480	21.69	6.74	5/4	KYDLFGYEVDTNTAPWIEK	−76	462	+	−	CC: mitochondria
			(20)	(5.78)		NCPPDLETYNATLQR	−75				
	precursor *Leishmania braziliensis*					IFEAPSKQDKPVDNESK	−92				
						ADGKEHPSALAQQQSLFEIK	−74				
						EHPSALAQQQSLFEIK	−89				
19	cytochrome c oxidase subunit V [*Leishmania infantum*]	XP_001470514	22.38	6,1	3/2	GWDNAALDTIYSSMLR	−90	278	+	−	BP: cellular metabolic process; CC: mitochondria; MF: electron carrier activity
			(28)	(5.78)		VFLPPHLGDPHR	−88				
						GAEIPDHVFQTPAVIER	−92				
26	60S acid ribosomal protein P2 *Leishmania braziliensis*	XP_001566999	10.65	4.38	3/3	AAGVAIELSR	−71	265	+	−	BP: Translational elongation; CC: ribosome; MF: Structural constituent of ribosome
			(13)	(4.6)		VDALFQELEGKSFDELM ^▴^ TEGR	−87				
						LVGSGSAAPAAAASTAGAAVAAAADAK	−85				

Proteins were extracted, submitted to 2D electrophoresis and identified by mass spectrometry using Mascot software and no redundant NCBI database.

**BP** = biological process; **CC = **cellular component; **ND** = not detected; **MF** = molecular function; **MW** = molecular weight; **p** ***I*** = isoelectric point; **p** = parts per million; ▴ = methionine oxidation; ▪ = acetylation.

## Discussion

Protozoan parasites, including *Leishmania*, depend on iron for survival and proliferation, as well as for the success of the colonization of the host. This means hosts should limit the parasite's access to the metal to counteract the infection. Although iron deprivation by chelating agents inhibits the growth of several protozoan parasites [15], [17]–[20], [29]–[31], the effect of iron chelation on *L. (V.) braziliensis* had not yet been addressed. In this paper, the effects of iron chelation on the growth, ultrastructure and protein expression of this parasite is described for the first time.

The proliferation and viability of *L. (V.) braziliensis* promastigotes was affected by both the concentration of the iron chelator and the time of exposure to iron-depleted medium. Parasite proliferation was completely inhibited after 24 h of exposure to 100 µM 2,2-dipyridyl. At this concentration, after 3 days, we observed a complete loss of cell viability. A possible explanation for the decrease in proliferation and viability may be related to the lack of protoporphyrin IX needed for tetrapyrrole biosynthesis. In fact, *Leishmania* spp. as well as other trypanosomatids are defective in several enzymes of the heme biosynthesis pathway and require exogenous sources of protoporphyrin IX or heme to sustain their viability [32]–[34]. Chelation of iron from the hemoglobin in the culture medium by 2,2-dipyridyl could provoke loss of the protoporphyrin IX required for tetrapyrrole biosynthesis, which could induce a defect in the electron transport respiratory complexes.

The inhibition of *Leishmania* spp. proliferation by iron chelators seems to rely also on the decrease of the activity of crucial enzymes. The iron chelator quercetin down-regulates the activity of ribonucleotide reductase, a Fe-dependent enzyme, and inhibits the proliferation of *L. (L.) donovani* amastigotes *in vivo* [35]. This enzyme catalyzes the rate-limiting step in DNA synthesis in these parasites, directly affecting their proliferation. A decrease in the activity of ribonucleotide reductase is also observed after treatment of tumor cells with iron chelators [6]. In the present study, 2,2-dipyridyl might have prevented the incorporation of cellular iron into newly synthesized iron-dependent proteins, including enzymes involved in DNA synthesis, resulting in the inhibition of *L. (V.) braziliensis* promastigote proliferation.

Iron deprivation induces mitochondrial dysfunction in various cell types. Ultrastructural analysis of lymphocytes, macrophages and reticular cells from the spleen of rabbits fed a low-iron diet has revealed mitochondrial swelling and loss of cristae and matrix of the organelle [36]. Similar changes are observed in the ultrastructure of lymphocytes from peripheral blood of anemic humans [37]. Here, analysis of the ΔΨm and the ultrastructure of *L. (V.) braziliensis* after treatment with the iron chelator revealed drastic damage to the mitochondrion of the parasite. Incubation of promastigotes with TMRE and subsequent analysis by flow cytometry showed that treatment with the iron chelator dissipated Δm in a dose-dependent manner. Moreover, TEM analysis of promastigotes cultured in iron-depleted medium demonstrated that parasites presented major mitochondrial injury. Because iron is an essential element for cellular respiration (acting as a cofactor of the mitochondrial enzymatic complexes and aconitase), several mechanisms may be associated with mitochondrial damage in iron-depleted medium For example, Fe^2+^ and Fe^3+^ associate with S^2−^, forming Fe-S clusters that receive and donate electrons in electron transport chain and Krebs cycle [38]. Dissipation of ΔΨm and reduction of ATP synthesis are associated with a decrease in the expression of the Fe-S subunit from complex II of hepatic Chang cells after treatment with the iron chelator DFO [39]. Although there are no reports regarding the effect of iron deprivation on the ultrastructure and mitochondrial activity of *Leishmania* spp., inhibition of *L. (L.) donovani* mitochondrial enzymatic complexes II and III by specific inhibitors also results in reduced ATP synthesis, increased concentration of cytosolic Ca^2+^ and dissipation of the ΔΨm [40]. Thus, it is possible to suggest that iron deprivation in *L. (V.) braziliensis* promastigotes induces a reduction or complete inhibition of ATP synthesis, leading to mitochondrial swelling and dissipation of ΔΨm. In fact, in mammal cells, reduction of ATP synthesis may lead to the opening of the mitochondrial permeability transition pore that allows the entrance of water and solutes (K^+^, Mg^2+^ and Ca^2+^ ions), which culminates in mitochondrial swelling and ΔΨm dissipation [41].

Although the sequence of events described above may characterize one possible mechanism of the iron chelator's action, it is likely that some promastigotes did not suffer from these effects. In fact, some parasites treated for 48 h with the iron chelator partially recovered from the dissipation of ΔΨm. These results suggest that during treatment with the iron chelator, some parasites suffered growth arrest and died, most likely due to mitochondrial dysfunction, whereas others adapted and survived for at least 48 h. This hypothesis is reinforced by the observation that treated parasites recovered their proliferation after reinoculation in fresh Schneider's medium without the iron chelator. In accord with this possibility, it was recently demonstrated that iron depletion induces a cellular adaptation that ultimately modulates the differentiation of promastigote into amastigote forms through the regulation of H_2_O_2_ level [42]. In the present study, TUNEL and PI assays showed that parasites treated for 24 or 48 h with the iron chelator did not undergo DNA fragmentation or cell membrane permeabilization. These results are consistent with the considerations above, indicating that those promastigotes that die from iron depletion may undergo an incidental type of cell death [43], while others can adapt to the nutritional stress and survive. In addition, we cannot rule out that 2,2-dipyridyl is cytotoxic to the parasites that die. However, the mechanism involved in the cytotoxicity of this compound would seem to be distinct from the DNA cleavage described in the literature [44].

The inhibition of proliferation and the ultrastructural alterations suffered by *L. (V.) braziliensis* promastigotes cultivated in iron-depleted medium were followed by changes in protein expression. According to the functional classification obtained from gene ontology terms, three down-regulated proteins (chain eukaryotic initiation factor 5A, 60S acid ribosomal protein P2 and mitochondrial ribonucleoprotein p18 precursor) are involved in the metabolism of nucleic acids, specifically by binding to RNA during translational elongation. Down-regulation of these proteins in iron-depleted conditions could be associated with inhibition of promastigote proliferation. In addition, down-regulation of proteins localized in the mitochondrion, such as p18 ribonucleoprotein mitochondrial precursor and cytochrome *c* oxidase subunit V, could be associated with the ultrastructural damage that this organelle suffered in the absence of iron. Cytochrome *c* oxidase, also known as complex IV, transfers electrons from cytochrome *c* to molecular oxygen in the mitochondrial respiratory chain. This enzymatic complex has heme as a cofactor and therefore depends directly on iron. In the present study, chelator-treated promastigotes showed a small decrease in the expression of cytochrome *c* oxidase, which may have contributed to the mitochondrial dysfunction observed. Thus, the ultrastructural damage observed in the mitochondrion could have been a consequence of iron chelation from the enzymes of the mitochondrial respiratory chain as well as of the down-regulation of other mitochondrial proteins. The proteins ubiquitin-conjugated enzyme E2 and ubiquitin-conjugating enzyme-like protein were down-regulated after the treatment with the iron chelator. The ubiquitin-proteasome system is a cytosolic multi-component machinery that selectively degrades proteins [45], [46]. Our results suggest that the down-regulation of these enzymes could be associated with the decrease of general metabolism of promastigotes submitted to iron deprivation. However, the role of iron depletion in the regulation of ubiquitin-conjugated enzyme E2 proteins remains to be elucidated.

Two down-regulated hypothetical proteins were identified in promastigotes cultivated in iron-depleted conditions. Concerning the hypothetical protein XP_001467133, no function could be assigned from the databases we searched. On the other hand, the hypothetical protein XP 001563170, identified in spot 14, was found to contain domains characteristic of proteins from the ALBA family. Proteins from the ALBA family bind to double-stranded DNA, rRNA and mRNA and may have a role in maintaining the structural and functional stability of RNAs and ribosomes. ALBA proteins are located in the cytoplasm of *T. brucei* and aggregate in mRNA-containing granules upon nutritional deprivation [47]. In addition, these proteins regulate the development of *T. brucei* in the insect vector and participate in the differentiation of the parasite [47]. Moreover, depletion of ALBA proteins reduces the initiation of protein translation and induces parasite growth arrest [48]. Thus, the down-regulation of this hypothetical protein XP 001563170, which contains ALBA domains, is in agreement with the general decrease of protein synthesis in the parasites treated with the iron chelator.

Calmodulin A was also down-regulated in iron-depleted promastigotes. Calmodulin is a key axonemal calcium sensor closely associated with ciliary motility and signaling processes in eukaryotes [49]. In *T. brucei*, another trypanosomatid, the down-regulation of this protein results in growth arrest and loss of cell viability [50]. Also in *T. brucei*, calmodulin is located in the flagellum and possibly takes part in the movement of the microtubules from the axoneme during the flagellar beat cycle [51]–[53]. Recycling of the transferrin receptor to the cell surface and exocytosis of transferrin [54] are calmodulin-dependent events, demonstrating a direct relationship between iron homeostasis and the expression of this protein. Decreased expression of calmodulin in the absence of iron could have led to the loss of motility and cell viability observed here in *L. (V.) braziliensis*, and it could have resulted in the loss of iron homeostasis in the promastigotes. When the transcript levels of differentially expressed proteins were analyzed, no differences were observed between treated and control parasites, confirming the poor correlation between mRNA and protein levels in *Leishmania* [55], [56].

Although the complexity of the mechanisms regulated by iron in *Leishmania* spp. are not completely understood, our results show that iron depletion leads to a multifactorial response that results in cellular collapse, starting with the interruption of cell proliferation and culminating in marked mitochondrial impairment in some parasites and their subsequent cell death, whereas others may survive and resume proliferating. Although iron is a crucial element to the biosynthesis of mitochondrial complexes, the parasites' survival in the absence of iron suggests a plasticity or resilience of the mitochondrion, as observed in other trypanosomatids under different environmental conditions [57], [58]. We suggest that, as has been proposed for *T. cruzi* and *T. brucei* submitted to different experimental conditions, other energetic pathways, such as glycolysis, could be accentuated in *Leishmania* after treatment with 2,2-dipyridyl to compensate for the mitochondrial dysfunction; however, this hypothesis must be further investigated in these parasites.

## Supporting Information

Figure S1 **Close-up of the gel regions showing invariant proteins.** (A–D) close-ups of different regions of the gels showing invariant proteins in both control and treated parasites. Each bar in the histograms represents the intensity means ± S.D. of gels from three independent experiments.
(TIF)

Figure S2 **mRNA expression levels in control and treated** ***L. (V.) braziliensis*** **promastigotes.** *EIF5A*, *CAL*, *UCEE2*, *UCELP*, *RP18* and *60S* mRNA expression levels were measured by qPCR. The values are expressed as normalized ratios of the target gene expression to the endogenous control, actin. Student's T test was used to compare promastigotes grown in control and iron-depleted medium.
(TIF)

Table S1 **Primers used for qPCR assays.**
(DOCX)

Table S2 **Proteins from** ***L. (V.) braziliensis*** **identified as equally expressed in both control and chelator-treated medium.** The degree of differential expression is shown in the histograms, presented as a grouped bar chart with error bars. Each bar represents the intensity means ± S.D. of gels from three independent experiments.
(DOCX)
