# Supplementary figures and images for: Cellular Growth and Mitochondrial Ultrastructure of Leishmania (Viannia) braziliensis Promastigotes Are Affected by the Iron Chelator 2,2-Dipyridyl
Source: PLoS Negl Trop Dis. 2013 Oct 17;7(10):e2481. doi: 10.1371/journal.pntd.0002481 (PMC3798463; doi:10.1371/journal.pntd.0002481)

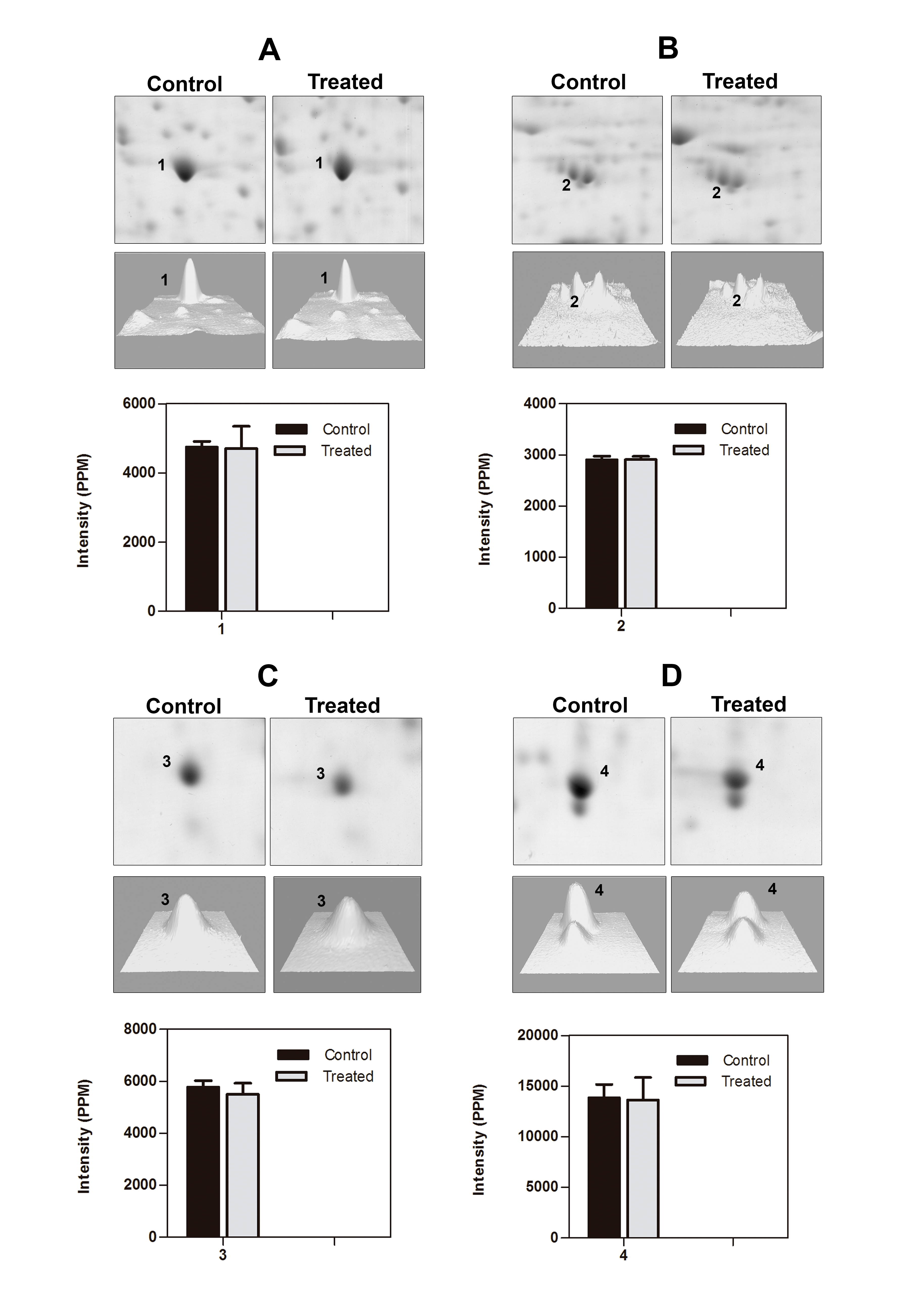

Supplement: Figure S1 — Close-up of the gel regions showing invariant proteins. (A–D) close-ups of different regions of the gels showing invariant proteins in both control and treated parasites. Each bar in the histograms represents the intensity means ± S.D. of gels from three independent experiments. (TIF) [file pntd.0002481.s001.tif]

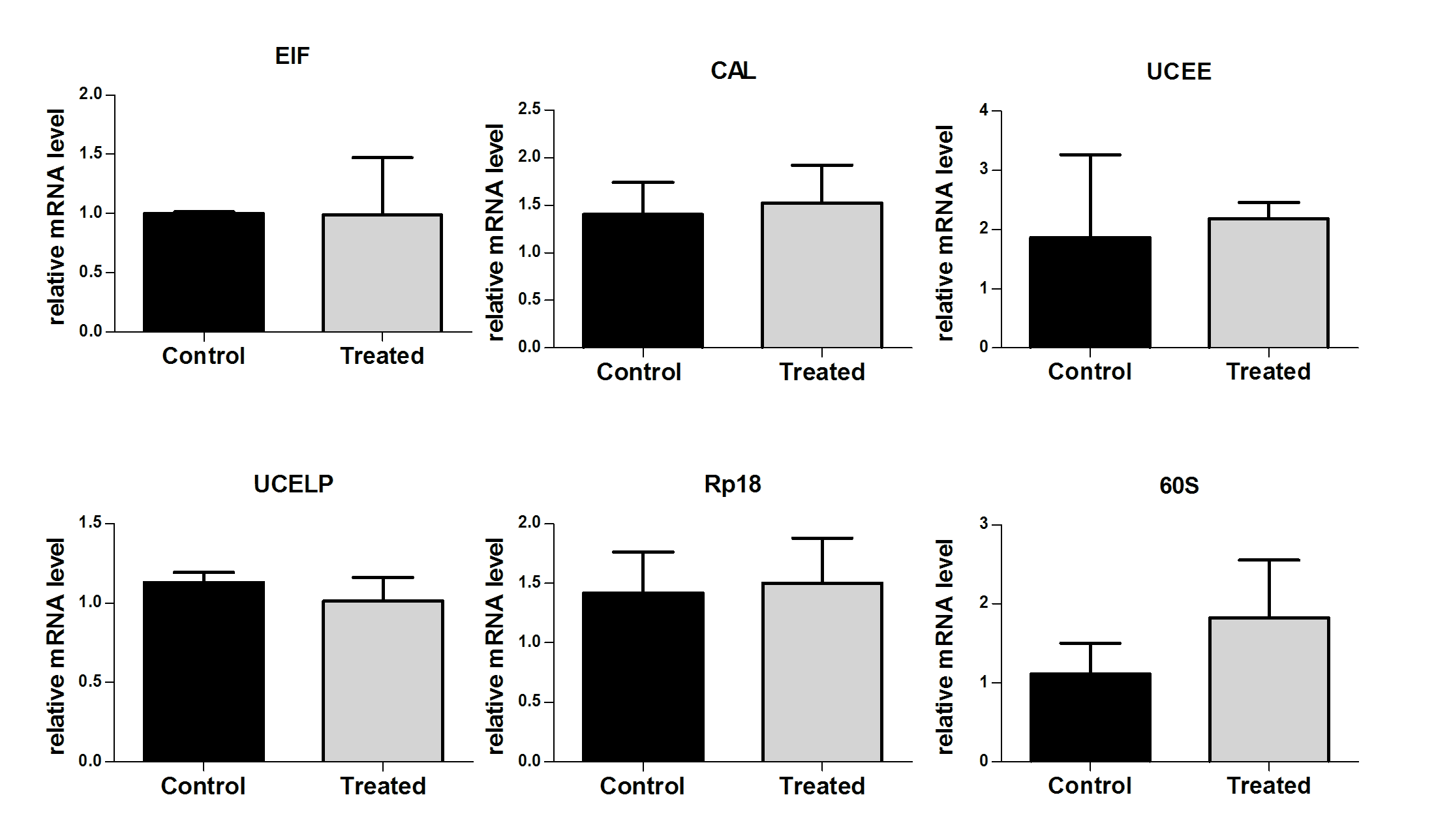

Supplement: Figure S2 — mRNA expression levels in control and treated L. (V.) braziliensis promastigotes. EIF5A, CAL, UCEE2, UCELP, RP18 and 60S mRNA expression levels were measured by qPCR. The values are expressed as normalized ratios of the target gene expression to the endogenous control, actin. Student's T test was used to compare promastigotes grown in control and iron-depleted medium. (TIF) [file pntd.0002481.s002.tif]
