# Supplementary material for: Cellular Growth and Mitochondrial Ultrastructure of Leishmania (Viannia) braziliensis Promastigotes Are Affected by the Iron Chelator 2,2-Dipyridyl
Source: PLoS Negl Trop Dis. 2013 Oct 17;7(10):e2481. doi: 10.1371/journal.pntd.0002481 (PMC3798463; doi:10.1371/journal.pntd.0002481)
Supplement: Table S1 — Primers used for qPCR assays. (DOCX) [file pntd.0002481.s003.docx]

**Table S1. Primers used for qPCR assays.**

| **Gene** | **Forward Primer** |  | **Reverse Primer** |
| --- | --- | --- | --- |
| EIF 5A | 5'-GATACGTGTGCATCAACGGC-3' |  | 5'-TGAAGATGTCGGTCGCAACA-3' |
| CAL | 5'-CTCTTTGACAAGGACGGCGA-3' |  | 5'-TCCTGGTCCACCTCGTTGAT-3' |
| UCEE 2 | 5'-TACCCGTTCAAGCCACCAAA-3' |  | 5'-GCACACGGAGAGTAACACCT-3' |
| UCELP | 5'AAAACGTCTCCGTGGGTCTG-3' |  | 5'-GCGACAGGATGCGATACTCA-3' |
| RP18 | 5'-AGCCAGCTTATCTGCACCG-3' |  | 5'-GCCGAACAGGTCGTACTTCT-3' |
| 60S | 5'-GGTGTTGCCATTGAGTTGTCC-3' |  | 5'-AGCTCATCGAAGCTCTTGCC-3' |
| ACT | 5’-GGCGAACGAGGAGTCATTTG-3’ |  | 5’-ATCAGCGACGGCTTGAACAG-3’ |
