# Supplementary material for: Cellular Growth and Mitochondrial Ultrastructure of Leishmania (Viannia) braziliensis Promastigotes Are Affected by the Iron Chelator 2,2-Dipyridyl
Source: PLoS Negl Trop Dis. 2013 Oct 17;7(10):e2481. doi: 10.1371/journal.pntd.0002481 (PMC3798463; doi:10.1371/journal.pntd.0002481)
Supplement: Table S2 — Proteins from L. (V.) braziliensis identified as equally expressed in both control and chelator-treated medium. The degree of differential expression is shown in the histograms, presented as a grouped bar chart with error bars. Each bar represents the intensity means ± S.D. of gels from three independent experiments. (DOCX) [file pntd.0002481.s004.docx]

**Table S2. Proteins from *L. (V.) braziliensis* identified as equally expressed in both control and chelator-treated medium.**

| **Code** | **Protein Name** | **NCBI Accession Nº** | **Theor. MW (Exp. MW)** | **Theor. p*I* (Exp p*I*)** | **Matching pep. / Pep. identified by MS/MS** | **Pep Sequence** | **Error ± ppm** | **Protein Score** | **Expression in control medium** | **Expression in chelator-treated medium** | **BP, CC and MF** |
| --- | --- | --- | --- | --- | --- | --- | --- | --- | --- | --- | --- |
| 1 | S- Adenosylhomocysteine hydrolase [*Leishmania donovani*] | AAA29265 | 48.44 | 5.75 | 9/5 | FDNLYGC^▲^R | -46 | 192 | = | = | BP: S-adenosylhomocysteine degradation; MF: Catalytic activity |
|  |  |  | (47.9) | (5.94) |  | EHVEIKPQVDR | -52 |  |  |  |  |
|  |  |  |  |  |  | TC^▲^C^▲^VC^▲^GYGDVGK | -48 |  |  |  |  |
|  |  |  |  |  |  | SKFDNLYGC^▲^R | -39 |  |  |  |  |
|  |  |  |  |  |  | QAEYINBPVDGPFKPDHYR | -39 |  |  |  |  |
| 2 | PDI, protein disulfide isomerase *Leishmania* *braziliensis* | XP_001569341.1 | 52.8 | 5.07 | 3/3 | EMEEFVR | -21 |  | = | = | BP: Cell redox homeostasis; CC: Endoplasmic reticulum |
|  |  |  | (50.3) | (4.81) |  | FYAPWC^▲^GHC^▲^K | -30 |  |  |  |  |
|  |  |  |  |  |  | YAPWC^▲^GHC^▲^QK | -30 |  |  |  |  |
| 3 | IgE-dependent histamine-releasing factor, putative. *Leishmania major* | XP_001683668 | 19.57 | 4.39 |  | VVDVVHNNR | -19 |  | = | = | ND |
|  |  |  | (23.0) | (4.42) |  | AFQANAAAFVK | -35 |  |  |  |  |
|  |  |  |  |  |  | ASYMAHIR | -21 |  |  |  |  |
| 4 | Calpain-like cysteine peptidase, putative *Leishmania braziliensis* | XP_001563372 | 13.16 | 4.73 |  | YENAQPTYSGNTVVK | -47 |  | = | = | BP: Endopeptidase activity; CC: Endopeptidase activity |
|  |  |  | (14.7) | (4.48) |  | DNGDGLLFR | -49 |  |  |  |  |
|  |  |  |  |  |  | ISFEANPIPK | -44 |  |  |  |  |

BP = Biological Process; CC = Cellular Component; MF = Molecular Function; MW = Molecular Weight; ND = not detected; p*I* = isoelectric point; ppm = parts per million;

▲ = carbamidomethylation of cysteine.
